# Effects of gadolinium contrast agent on aortic blood flow and myocardial strain measurements by phase-contrast cardiovascular magnetic resonance

**DOI:** 10.1186/1532-429X-12-70

**Published:** 2010-11-24

**Authors:** Erik Hedström, Karin Markenroth Bloch, Erik Bergvall, Freddy Ståhlberg, Håkan Arheden

**Affiliations:** 1 Department of Clinical Physiology, Lund University, Lund, Sweden; 2 Philips Medical Systems, Best, the Netherlands; 3 Department of Imaging and Function, Skåne University Hospital, Lund, Sweden; 4 Department of Medical Radiation Physics, Lund University, Lund, Sweden; 5 Department of Radiology, Lund University, Lund, Sweden

## Abstract

**Background:**

Quantitative blood flow and aspects of regional myocardial function such as myocardial displacement and strain can be measured using phase-contrast cardiovascular magnetic resonance (PC-CMR). Since a gadolinium-based contrast agent is often used to measure myocardial infarct size, we sought to determine whether the contrast agent affects measurements of aortic flow and myocardial displacement and strain. Phase-contrast data pre and post contrast agent was acquired during free breathing using 1.5T PC-CMR.

**Results:**

For aortic flow and regional myocardial function 12 and 17 patients were analysed, respectively. The difference pre and post contrast agent was 0.03 ± 0.16 l/min for cardiac output, and 0.1 ± 0.5 mm for myocardial displacement. Linear regression for myocardial displacement (MD) after and before contrast agent (CA) showed MD_postCA _= 0.95MD_preCA_+0.05 (r = 0.95, p < 0.001). For regional myocardial function, the contrast-to-noise ratios for left ventricular myocardial wall versus left ventricular lumen were pre and post contrast agent administration 7.4 ± 3.3 and 4.4 ± 8.9, respectively (p < 0.001). The contrast-to-noise ratios for left ventricular myocardial wall versus surrounding tissue were pre and post contrast agent administration -16.9 ± 22 and -0.2 ± 6.3, respectively (p < 0.0001).

**Conclusions:**

Quantitative measurements of aortic flow yield equal results both in the absence and presence of gadolinium contrast agent. The total examination time may thereby be reduced when assessing both viability and quantitative flow using PC-CMR, by assessing aortic flow post contrast agent administration. Phase-contrast information for myocardial displacement is also assessable both in the absence and presence of contrast agent. However, delineation of the myocardium may be difficult or impossible post contrast agent due to the lower image contrast. Acquisition of myocardial displacement should therefore be performed pre contrast agent using current PC-CMR sequences.

## Background

Quantitative assessment of cardiac function and blood flow is of importance for diagnosis and evaluation of treatment in patients with cardiac disease. Aspects of regional function such as motion and myocardial displacement can be measured using phase-contrast cardiovascular magnetic resonance (PC-CMR). Myocardial strain can then be calculated from myocardial displacement[1].

Multiple studies have validated PC-CMR for noninvasive flow measurements and shown a high degree of accuracy[2-4]. Important parameters when using PC-CMR for noninvasive flow measurements have been suggested to be acquisition dependent parameters such as spatial resolution[5,6] and slice orientation, as well as post processing parameters as ROI size and its correct ROI placement[7].

A gadolinium-based contrast agent is often administered during the CMR session for acquisition of myocardial infarct size. Thus, it is of importance to know whether presence of contrast agent has impact on flow quantification using PC-CMR data, by for instance altered signal-to-noise (SNR) ratio and/or additional phase effects owing to the contrast agent administration. Small vessels have been shown to be more easily delineated when adding a contrast agent[8]. Delineating and evaluating myocardium and differentiating it from the blood pool and surrounding tissue may however be more difficult. This can be expected since the gadolinium-based contrast agent affects magnitude images both regarding intensity and contrast, and also by increased sensitivity to flow artefacts. To our knowledge, an in vivo investigation of PC-CMR accuracy of quantification after contrast agent administration has not been previously performed.

We therefore sought to investigate whether the presence of contrast agent affects the phase map acquired by PC-CMR, and thereby quantitative measurements of blood flow in the aorta and regional myocardial displacement of the left ventricle in humans.

## Materials and methods

The protocol and procedures complies with the Declaration of Helsinki, and were approved by the local research ethics committee. All patients gave their written informed consent to participate.

The study population consisted of prospectively included patients with a first-time myocardial infarction. For measurement of quantitative flow in the ascending aorta and myocardial regional function, respectively, a population of 14 (13 men, median age 62 years, range 41-71) and 17 (14 men, median age 62 years, range 41-73) patients were included for the study. All patients from the first group were also included in the regional function acquisition group.

To determine quantitative flow in the aorta and left ventricular myocardial displacement, PC-CMR was performed using a 1.5 T Intera CV scanner and a 5-element cardiac synergy coil (Philips Medical Systems, Best, the Netherlands), with and without the administration of an extracellular contrast agent (0.2 mmol/kg, gadoteric acid, Gd-DOTA, Guerbet, Gothia Medical AB, Billdal, Sweden). The protocol consisted of acquisition of phase contrast data in left ventricular longaxis views followed by a transversal slice through the ascending aorta. Thereafter the contrast agent was administered and a perfusion sequence was executed during the maximum breath-hold time possible for each patient. After free breathing for five minutes, the phase-contrast data acquisition was repeated. All sequences were acquired during free breathing. To compare the measurements of flow in the aorta and left ventricular regional function pre and post contrast agent administration, cardiac output and myocardial displacement was derived from the phase-contrast data, respectively.

### PC-CMR acquisition

Quantitative flow measured by PC-CMR is directly proportional to the image intensity in the phase-contrast data set, since the phase shift utilized for measurements using PC-CMR is proportional to the velocity of the moving spin in a setting with linear field gradients[9]. Flow was thus calculated as mean velocity (cm/s) in each ROI multiplied by the ROI area (cm^2^).

For aortic flow, through-plane phase-contrast data was acquired perpendicular to the ascending aorta at the level of the pulmonary trunk bifurcation. For myocardial displacement, phase-contrast data was acquired in two in-plane directions simultaneously in long-axis images (3-, 4- and 2-chamber views) to obtain a pseudo-3D coverage of the left ventricle. Both data sets were acquired pre and post contrast agent administration. All scans were retrospectively triggered on the R-peak. For aortic flow and myocardial displacement respectively, 35 and 22 time frames were acquired over the cardiac cycle, and the acquisition times were approximately 1 min and 1.5 min. For aortic flow this gave a time resolution between 17 and 37 ms, and for myocardial displacement a reconstructed temporal resolution of 45 ms. Saturation bands (30 mm thickness, 30 mm gap to image plane) on both sides of the imaging plane were applied to reduce signal from blood during sampling for myocardial displacement[10]. Spatial resolution, matrix size, FOV, TR/TE, and VENC was for aortic flow 1.4 × 1.4 × 6 mm^3^, 256 × 256, 350 × 350 mm^2^, 8/5 ms, and 2.0 m/s, and for myocardial displacement 1.6 × 1.6 × 8 mm^3^, 256 × 192, 400 × 300 mm^2^, 23/5 ms, and 0.2 m/s. The flip angle was 15°. All data was collected using a gradient-echo pulse sequence.

### Quantitative aortic flow measurement

**Figure 1:**
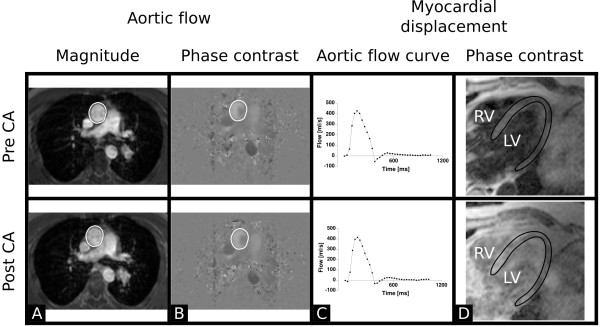
**Representative cases for aortic magnitude images (A), aortic phase contrast images (B), aortic flow curves (C), and myocardial displacement magnitude images (D)**. The upper and bottom row respectively represents pre and post contrast agent data. Please note that no visual differences are found pre and post contrast agent administration in A-C. In **A **and **B**, the ROI delineating the aorta is indicated. In **D**, the lower image contrast between myocardium and blood pool and surrounding tissue post contrast agent administration is shown. Due to the lower image contrast, delineation of the left ventricular myocardium may be difficult post contrast agent administration. The left ventricular myocardial wall is delineated as performed by the automatic algorithm in the pre contrast agent image. The delineation has been copied onto the post contrast agent image for comparison. CA = contrast agent; LV = left ventricle; RV = right ventricle.

Vessel delineation was performed by two experienced observers (KMB, EH) using the free program Segment v1.8R0839[11]. For aortic flow measurements, a circular ROI was manually placed over the ascending aorta in the end-diastolic pre contrast agent magnitude image. This ROI was deformed to match the area of the aorta, and propagated automatically throughout the cardiac cycle. Manual correction was applied when the ROI did not match the aorta. Information in the magnitude and phase-contrast images was used for guidance of ROI placement and size. The phase-contrast images were used for delineation of the aorta where the flow had high contrast versus the surrounding tissue, approximately until the late systolic phase of the cardiac cycle. This was repeated for the post contrast agent images. For an example of delineation of the aorta, see Figure 1A-B. Since pre and post contrast agent administration measurements were performed with exactly the same protocol settings and image orientation, the background phase is expected to be unaltered and will therefore not influence the results from this study. Thus, no further background phase effects correction was performed.

### Myocardial displacement measurement

Myocardial displacement was evaluated by one observer (EH) using an automated method, developed in-house using Matlab R2007b (The Mathworks Inc, MA, USA). Phase-contrast data was integrated with respect to time to obtain the displacement field in the myocardium. Delineation of the left ventricular myocardium was performed manually in the first time-frame of each set of magnitude images acquired before contrast agent administration. The previously described automated and validated technique with a fitted spatiotemporal motion model was used for propagation throughout all time-frames[1,12]. The mask was then transferred to the PC images acquired pre and post contrast agent administration for measurement of myocardial displacement. The Cauchy-Green strain tensor was calculated by numerical differentiation of the displacement field. End diastole was used as reference configuration (zero displacement). The myocardial displacement at end systole was calculated pixelwise by integration of velocity with respect to time. An example of pre and post contrast agent magnitude images for measurement of myocardial displacement is shown in Figure 1D. A single maximum and average myocardial displacement value was respectively calculated in each patient for images acquired pre and post contrast agent administration.

### Signal-to-noise and contrast-to-noise

The signal-to-noise (SNR) and contrast-to-noise (CNR) ratios were calculated as follows. For aortic SNR, the mean value of signal within the ROI representing the aorta was divided by the standard deviation of noise measured in a large ROI outside the torso. For the aorta ROI signal measurement, the cardiac time phase with the highest signal was chosen. For CNR in the myocardial displacement images, myocardial wall SNR was subtracted from the SNR for left ventricular lumen and SNR for surrounding tissue, respectively. The signal for myocardium, lumen and surrounding tissue was divided by the standard deviation of noise measured as described above. The placement of the ROI for assessing noise was in all cases such as to avoid ghosting and aliasing artefacts.

### Statistical Analysis

Statistical analyses were performed using Matlab R2007b (The Mathworks Inc, MA, USA). Data is presented as mean ± SD. Agreement between pre and post contrast agent phase-contrast measurements as well as interobserver variability were analyzed using the Bland-Altman method. Linear regression and comparison and confidence intervals (CI) were calculated for myocardial displacement. Comparison of datasets pre and post contrast agent administration was performed with a two-tailed Student's t test. A p value < 0.05 was considered statistically significant.

## Results

### Aortic flow measurement

In 2 patients, background correction failed in either pre (2 cases) or post (2 cases) contrast agent acquisitions. The reason for this was unknown. Background correction however worked after restarting the scanner. At that time point contrast agent was already administered and thus these patients were excluded from further analysis.

The time between pre and post contrast agent aortic flow acquisitions was 14 ± 4 min. The paired absolute difference in heart rate pre and post contrast administration was 0 min^-1 ^to 6 min^-1^.

The ROI areas were not different in size pre and post contrast agent administration (1% ± 5%; p = 0.55). The SNR was generally higher post contrast agent administration, but not significant (20% ± 20%; p = 0.08).

The difference in cardiac output pre and post contrast agent administration was 0.03 ± 0.16 l/min for observer 1, and 0.12 ± 0.24 l/min for observer 2. A weak correlation was found between differences in cardiac output and heart rate pre and post contrast agent administration (r = 0.37, p = 0.04).

Interobserver variability for cardiac output was -0.03 ± 0.08 l/min and 0.02 ± 0.15 l/min pre and post contrast agent, respectively. The ROI was equally often manually corrected in pre and post contrast agent aortic flow images. Aliasing indicating wrapping of velocity encoding was not found within the ROI in any image. The velocity offset error was below 0.6 cm/s in all evaluated patients[13].

### Myocardial displacement measurement

**Figure 2:**
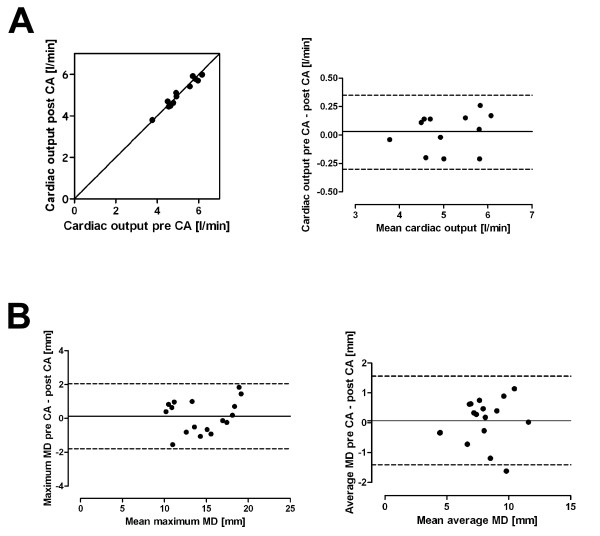
**Cardiac output and myocardial displacement pre and post contrast agent acquisition**. **A)** Agreement shown with line of identity (left) of cardiac output between pre and post contrast agent acquisitions. Bland-Altman plot (right) showing differences between pre and post contrast agent acquisitions (mean and 2SD indicated; mean 0.03 l/min, limits of agreement -0.30-0.35 l/min). Measurements by observer 1 are plotted. **B) **Bland-Altman plots showing differences between myocardial displacement for maximum (left; mean 0.13 mm, limits of agreement -1.79-2.05 mm) and average (right; mean 0.07 mm, limits of agreement -1.41-1.56 mm) values pre and post contrast agent administration (mean and 2SD indicated). CA = contrast agent; MD = myocardial displacement.

No data was excluded from this analysis. The time between corresponding long axis chamber views pre and post contrast agent (CA) was 14 ± 2 min. The heart rate differences were equivalent to the differences found during aortic flow measurements. Linear regression showed that myocardial displacement (MD) was MD_postCA _= 0.95MD_preCA _+ 0.05 (r = 0.95, p < 0.001). The 95% CI were for the slope and y intercept 0.93-0.98 and 0.01-0.08, respectively. The slope was not significantly different from 1 (p = 0.52) and the offset for the y intercept was not significantly different from 0 (p = 0.57). Bland-Altman analysis showed that MD_postCA _was 0.1 ± 0.5 mm shorter than MD_preCA_. No systematic difference was detected. The single maximum (p = 0.59) and average (p = 0.69) myocardial displacement measurements were not significantly different between images acquired pre and post contrast agent administration (Figure 2B).

As visualised in Figure 1D, the image contrast between myocardium and blood pool and surrounding tissue was lower in the post contrast agent magnitude images compared to the pre contrast agent magnitude images. The CNR for left ventricular myocardial wall versus left ventricular lumen were pre and post contrast agent administration 7.4 ± 3.3 and 4.4 ± 8.9, respectively (p < 0.001). The CNR for left ventricular myocardial wall versus surrounding tissue were pre and post contrast agent administration -16.9 ± 22 and -0.2 ± 6.3, respectively (p < 0.0001). The delineation of myocardium for myocardial displacement measurements was only performed in images acquired pre contrast agent administration, since the automatic algorithm did not work in images acquired post contrast agent administration due to low image contrast.

## Discussion

Our study shows that quantitative aortic flow can be assessed equally well by delineation in both pre and post contrast agent magnitude images, and the PC images may be used for improved delineation guidance. Hence, possible phase effects, caused by e.g. altered post contrast magnitude signal in the flowing compartment of edge pixels were small. For measurement of myocardial displacement, it is also feasible to assess information from phase-contrast images both pre and post contrast agent. However, the lower contrast in magnitude images post contrast agent administration makes it difficult to automatically and correctly delineate the myocardial wall for acquisition of myocardial displacement using the current PC-CMR sequences.

The interobserver variability for aortic flow in the present study was in accordance with a previous study in man[4]. It is likely that individual differences in cardiac output between measurements pre and post contrast agent are related to physiological phenomena. In the 2 patients excluded due to failure of automatic background correction, the phase background may be manually corrected and thus it is still possible to provide a clinically relevant examination. This manual correction was however not performed in the present study.

Previously, gadolinium-based contrast agents have been used for introducing phase contrast changes for assessing blood oxygenation[14] and arterial input functions[15]. In these settings, a bolus dose is given. The magnetic field perturbations introduced with the addition of contrast agent might be hypothesised to incur alterations also on the measured velocities using PC-CMR for flow quantification. In the present study, however, measurements were made not during a bolus, but rather during a pseudo steady state, and we did not find any introduced phase-contrast changes under these circumstances. It can not from the present results, however, be ruled out that acquisition of phase-contrast data for quantitative flow very early after contrast agent administration, could result in erroneous measurements due to bolus effects. The quality of aortic flow data images may also be improved by using a contrast agent, due to increased SNR. Thus, delineation of the aorta may be simplified in the presence of contrast agent. In the present study, however, the aorta was found to be easily delineated in both absence and presence of contrast agent, and no effect was found from increased SNR.

The lack of systematic differences in myocardial displacement measurements pre and post contrast agent suggests that presence of contrast agent does not affect measurement of myocardial displacement as such, nor induces image artifacts in the PC images. Therefore, myocardial strain may be calculated also in the presence of contrast agent. Major manual corrections are however needed to delineate the left ventricular wall using the present protocol, due to lower magnitude image contrast post contrast agent administration. The manual delineation is very time consuming. The low image contrast is also likely to have negative effects on accuracy since the blood pool or pericardial structures may falsely be included as myocardium.

## Study limitations

In this study, we did not optimise sequence parameters previously reported by others to be significant to the results such as slice thickness and orientation, or spatial resolution[5]. Instead, we used a phase-contrast sequence commonly used for clinical applications. The myocardial displacement acquisitions were performed in-plane in two dimensions and thus did not consider the full myocardial kinetics in three dimensions. However, long-axis slices were used to reduce the impact of out-of-plane motion, since it is assumed that typical myocardial torsion values are on the order of the slice thickness or less. All two-dimensional methods suffer from this, but with recently developed CMR sequences acquiring three-dimensional data, this may be fully studied in the future.

Few females were included and therefore the results may not be generalized over genders. It is however highly unlikely that measurement of velocity by PC-CMR should differ between genders.

## Conclusions

Acquisition of aortic flow is feasible both in the absence and presence of contrast agent. This implies that the total examination time can be reduced when assessing both viability and quantitative blood flow using CMR, since blood flow can be assessed after contrast agent administration.

Even though myocardial displacement is assessable both in the absence and presence of contrast agent, delineation of the myocardium after contrast agent administration may be difficult and more time-consuming due to the lower image contrast between myocardium and blood pool and surrounding tissue. In our opinion, acquisition of myocardial displacement using the current PC-CMR sequences should therefore currently be performed pre contrast agent administration.

## Competing interests

The authors declare that they have no competing interests.

## Authors' contributions

EH designed the study, acquired, analysed and interpreted data, and drafted the manuscript. KMB analysed and interpreted data, and revised the manuscript. EB designed the study, interpreted data, and revised the manuscript. FS interpreted data and revised the manuscript. HA designed the study, interpreted data, and revised the manuscript. All authors read and approved the final manuscript.
